# Evaluation of Tp-e interval, Tp-e/QT ratio and Tp-e/QTc ratio in patients with acute myocarditis

**DOI:** 10.1186/s12872-019-1207-z

**Published:** 2019-10-22

**Authors:** Fatih Mehmet Ucar, Cihan Ozturk, Mustafa Adem Yılmaztepe

**Affiliations:** 1 Department of Cardiology, Demiroğlu Bilim University, İstanbul, Turkey; 2 Department of Cardiology, Trakya University Hospital, Edirne, Turkey

**Keywords:** Electrocardiography, Tp-e interval, Tp-e/QT ratio, Acute myocarditis

## Abstract

**Background:**

Acute myocarditis (AM) can be defined as an inflammatory disease of the myocardium and characterized by large heterogeneity of clinical presentation. Myocarditis is becoming increasingly recognized as a contributor to unexplained mortality, and is thought to be a major cause of sudden cardiac death in the first two decades of life. The present study aimed to search the assessment of repolarization dispersion measured from the 12-lead surface electrocardiogram (including Tp-e interval, Tp-e/QT and Tp-e/QTc ratios) in AM patients.

**Methods:**

Totally 56 patients (mean age was 22 ± 3.7 years and 67% of the patients were male) with AM and 56 control subjects (23 ± 4.7 years and 64% of the patients were male) were enrolled. Tp-e intervals, Tp-e/QT and Tp-e/corrected QT (QTc) ratios were calculated from 12-lead electrocardiogram.

**Results:**

Heart rate, QT and QTc values were similar between groups. QRS interval was lower in AM group compared to the control group (*p* <  0.001). Tp-e, Tp-e/QT and Tp-e/ QTc were significantly higher in AM group (*p* <  0.001, *p* <  0.001, *p* = 0.03 respectively) and they were significantly correlated with high troponin and high sensitive C reactive protein levels. In hospital follow-up time was 6 ± 2 days. Four patients have non sustained ventricular tachyarrhythmias and 1 patient dead because of cardiac arrest.

**Conclusions:**

Our study demonstrated that Tp-e intervals, Tp-e/QT and Tp-e/QTc ratios were higher in patients with AM than control subjects. The increased frequency of ventricular arrhythmias can be clarified by increased indexes of ventricular repolarization parameters in patients with AM.

## Background

Acute myocarditis (AM) is inflammation of the myocardium that manifests with different clinical presentations [1]. While the patients who present with chest pain generally have a better prognosis, patients presenting with heart failure have a higher risk of sudden cardiac death [2]. Ventricular arrhythmias (VA), including premature ventricular contractions, ventricular tachycardia (VT) or unheralded ventricular fibrillation (VF) are also frequently seen in myocarditis. Myocarditis can be the cause of sudden death, particularly in young people without structural heart disease [3–5].

QT dispersion, corrected QT dispersion and transmural dispersion of repolarization (DoR) were used for evaluation of myocardial repolarization. T peak-to-end (Tp-e) interval has been proposed as an index of total DoR [6, 7]. Furthermore, Tp-e interval, Tp-e/QT and Tp-e/corrected QT (QTc) ratios are also electrocardiographic indices, that can be calculated from 12 lead surface ECG, showing total DoR [7, 8]. Additionally, an increase in these parameters have been linked with VAs [9, 10]. The main objective of this study was to assess the dispersion of ventricular repolarization by measuring Tp-e interval, Tp-e/QT and Tpe/QTc ratios, in patients with AM.

## Methods

### Study population

The present study was a a single-centered study, based on retrospective analysis of the data collected in Trakya University Hospital in Turkey between January 2010 and November 2016. The database were screened for clinically suspected myocarditis based on the criteria defined by the working group of the European Society of Cardiology on myocardial and pericardial diseases [11]. All ECG parameter’s were collected from patients files at admission. Totally 56 patients with acute myocarditis and age and sex matched 56 control subjects were enrolled to the study. All control group patients were healthy volunteers and study consent form was signed from all subjects. Informed consent forms were obtained from all participants in writing. The study protocol was was conducted in accordance with The Declaration of Helsinki and approved by Trakya University Ethics Committee.

### Electrocardiography

At rest in the supine position the 12-lead ECG was recorded at a paper speed of 50 mm/s (Nihon Kohden, Tokyo, Japan). ECG taken during the patients evaluation and resting heart rate was calculated. QT and QRS intervals were measured manually with calipers and magnifying glass for decreasing the error measurements. ECG measurements of QT and QRS intervals were performed by two cardiologists who were blinded to the patient data. The measurements were performed on lead II and lead V5 and then the longest QT interval was selected for the analysis. The QT interval was measured from the beginning of the QRS complex to the end of the T wave and heart rate corrected QT interval (QTc) was calculated using the Bazett’s formula: From the peak of T wave to the end of T wave was defined as Tp-e interval. The interobserver and intraobserver coefficients of variation were found to be 2.4 and 2.7%, respectively.

### Echocardiography

Echocardiographic measurements of the patients were analysed by an experienced cardiologist in accordance with the American Society of Echocardiography (ASE) guidelines [12]. Vivid–7 (GE Vingmed, *Horten*, *Norway*) device was used for the examination and by using the modified Simpson’s method left ventricular ejection fraction was calculated.

### Laboratory

All the laboratory results were gathered from clinic database. Blood samples were taken after a 12- h fasting period, fasting blood glucose was measured using the hexokinase method. Blood sapmles for complete blood count were collected in Monovette tubes (Sarstedt, Leicester, United Kingdom) containing ethylenediamine tetraacetic acid anticoagulated. Leukocyte counts were calculated by an automated analyzer (Abbott Laboratory, Illinois, USA). Serum level of C*RP* was *measured* by using an Ary360 automatic rate turbidimetric system (Beckman Coulter, Inc., Brea, CA, USA). Cardiac troponin I (cTnI) was measured from lithium-heparinised plasma with AQT90 FLEX TnI immunoassay (Radiometer Medical ApS, Denmark). The upper 99th percentile upper reference limit has been determined as ≤0.023 μg/L.

### Statistical analysis

Continuous variables are expressed as a mean ± standard deviation or as median with interquartile range. Numbers and percentages were used for categorical variables. For comparing the categorical variables χ^2^ test or Fisher’s exact test was used. Data were tested for normal distribution using the Kolmogorov-Smirnov test, Student’s t-test or Mann-Whitney U test was used for continuous variables, when appropriate. Pearson’s correlation test was used for correlational analysis. All statistical analyses were performed using SPSS software version 17.0 (SPSS Inc., Chicago, IL). A *p*-value of < 0.05 was considered statistically significant.

## Results

The mean age of the subjects were 22.8 ± 4.2 years; the clinical characteristics and laboratory parameters of the subjects are given in Table 1. There were no differences between two groups, in terms of baseline demographic and clinical characteristics (all *p* > 0.05). White blood cell (WBC), *high-sensitivity C-reactive protein* (hsCRP), *Alanine transaminase* (ALT) and Aspartate aminotransferase (AST), levels were all higher in AM group (*p* <  0.001).

**Table 1:** Baseline demographic, clinical characteristics, and laboratory parameters of the study subjects

	Myocarditis Group (*n* = 56)	Control Group (*n* = 56)	*p*
Male (%,n)	67% (38)	64% (36)	0.69
Age	22 ± 3.7	23 ± 4.7	0.35
Hyperlipidemia (%,n)	10% [6]	3% [2]	0.14
Smoking (%,n)	14% [8]	5% [3]	0.11
Body Mass index	26.5 ± 3.4	26.5 ± 3.1	0.95
Systolic Blood Pressure (mmhg)	119 ± 12	123 ± 9	0.11
Diastolic Blood Pressure (mmhg)	75 ± 9	76 ± 8	0.57
Glucose (mg/dL)	99 ± 18.0	97 ± 17.5	0.58
Creatinine (mg/dL)	0.83 ± 0.12	0,80 ± 0.13	0.26
Na (mEq/L)	139 ± 2.5	140 ± 2.2	0.18
K (mEq/L)	4.4 ± 0.8	4.5 ± 0.3	0.78
Ca (mg/dL)	9.1 ± 0.4	9.0 ± 0.4	0.22
AST (mg/dL)	54 (9–159)	20 (12–39)	**< 0.001**
ALT (mg/dL)	28 (9–70)	20 (6–52)	**< 0.001**
LDL (mg/dL)	98 ± 30.9	108 ± 28.8	0.08
HDL (mg/dL)	37 ± 10.9	40 ± 9.1	0.09
hs-CRP (mg/dL)	4.16 ± 4.32	0,32 ± 0.15	**< 0.001**
Wight blood cell, × 109/L	9.7 ± 2.6	7.8 ± 1.9	**< 0.001**
Neutrophil, × 109/L	5.9 ± 0.50	6.6 ± 0.06	0,61
Lymphocyte, × 109/L	2.0 ± 0.25	1.5 ± 0.14	**0.01**
Hemoglobin (mg/dL)	14.5 ± 1.4	13.7 ± 1.3	0.74

*Na* Sodium, *K* Potassium, *Ca* Calcium, *AST* Aspartate aminotransferase, *AST Alanine transaminase, LDL* Low density lipoprotein*, HDL* High density lipoprotein*, CRP* C-reactive protein

AST-ALT-WBC-CRP AND Lymphocyte levels are boldface, because they are significant (*p* < 0.05)

The echocardiographic and electrocardiographic measurements are shown in Table 2. Left ventricular ejection fraction and left atrial diameter were similar in two groups (*p* = 0.11 and *p* = 0.13, consecutively). Heart rate, QTc and Tp-e intervals, Tp-e/QT and Tp-e/QTc ratios were all significantly higher in patients with AM compared to control subjects (*p* < 0.001, *p* < 0.001, *p* < 0.001, *p* < 0.001 and *p* < 0.03, respectively), additionally QRS duration was also significantly longer in AM group (*p* < 0.001). Four patients has arrhytmic events during hospitalization and electrocardiographic characteristics of myocarditis patients with or without arrhythmia during hospitalization are similiar (Table 3).

**Table 2:** Echocardiographic and electrocardiographic characteristics of the study population

	Myocarditis Group (*n* = 56)	Control Group (*n* = 56)	*p*
Ejection Fraction (%)	61 ± 7.7	63 ± 5.8	0.11
LVEDD (mm)	45 ± 4.8	46 ± 3.1	0.44
LVESD (mm)	30 ± 4.1	29 ± 3.9	0.36
LVEDV (mL)	191 ± 31,1	185 ± 31,7	187 ± 34,3
LVESV (mL)	127 ± 21,4	124 ± 24,1	125 ± 24,3
Left atrial diameter (mm)	31 ± 3.4	33 ± 3.9	0.13
LAa (cm2)	14.8 ± 3.3	15.0 ± 2.6	0.79
LAv (cm3)	39 ± 13.8	35 ± 8.8	0.08
Heart rate (bpm)	90 ± 19.3	75 ± 13.8	**< 0.001**
QT interval (ms)	370 ± 49.0	362 ± 30.2	0.33
QTc interval (ms)	437 ± 55.5	404 ± 32.5	**< 0.001**
QRS interval (ms)	118 ± 22.1	98 ± 10.0	**< 0.001**
Tp-e interval (ms)	85 ± 12.5	74 ± 7.3	**< 0.001**
Tp-e/QT ratio	0,23 ± 0.04	0,20 ± 0.02	**< 0.001**
Tp-e/QTc ratio	0,19 ± 0.03	0,18 ± 0.02	**0.03**

Data are represented as mean values ±SD. *LVEDD* Left Ventricle End Diastolic Diameter, *LVESD* Left Ventricle End Systolic Diameter, *LVEDV* Left ventricular end-diastolic volüme, *LVESV* Left ventricular end-systolic volüme, *LAa* Left atrial area, *LAv* Left atrial volume, *mm* millimeters, *bpm* beats per minute, *Tp-e interval* T-peak to T-end interval; c = rate corrected value; B = corrected with Bazett’s formula

AST-ALT-WBC-CRP AND Lymphocyte levels are boldface, because they are significant (*p* < 0.05)

**Table 3:** Electrocardiographic characteristics of myocarditis patients with or without arrhythmia during hospitalization

	Arrhythmia Patients (*n* = 4)	No Arrhythmia Patients (*n* = 52)	p
Heart rate (bpm)	101 ± 36	89 ± 17	0.27
QT interval (ms)	355 ± 41.2	371 ± 49.7	0.52
QTc interval (ms)	453 ± 91.1	436 ± 53.1	0.55
QRS interval (ms)	100 ± 28.2	119 ± 21.2	0.08
Tp-e interval (ms)	95 ± 10.0	84 ± 12.4	0.11
Tp-e/QT ratio	0,27 ± 0.05	0,23 ± 0.04	0.07
Tp-e/QTc ratio	0,21 ± 0.04	0,19 ± 0.03	0.35

**Fig. 1:**
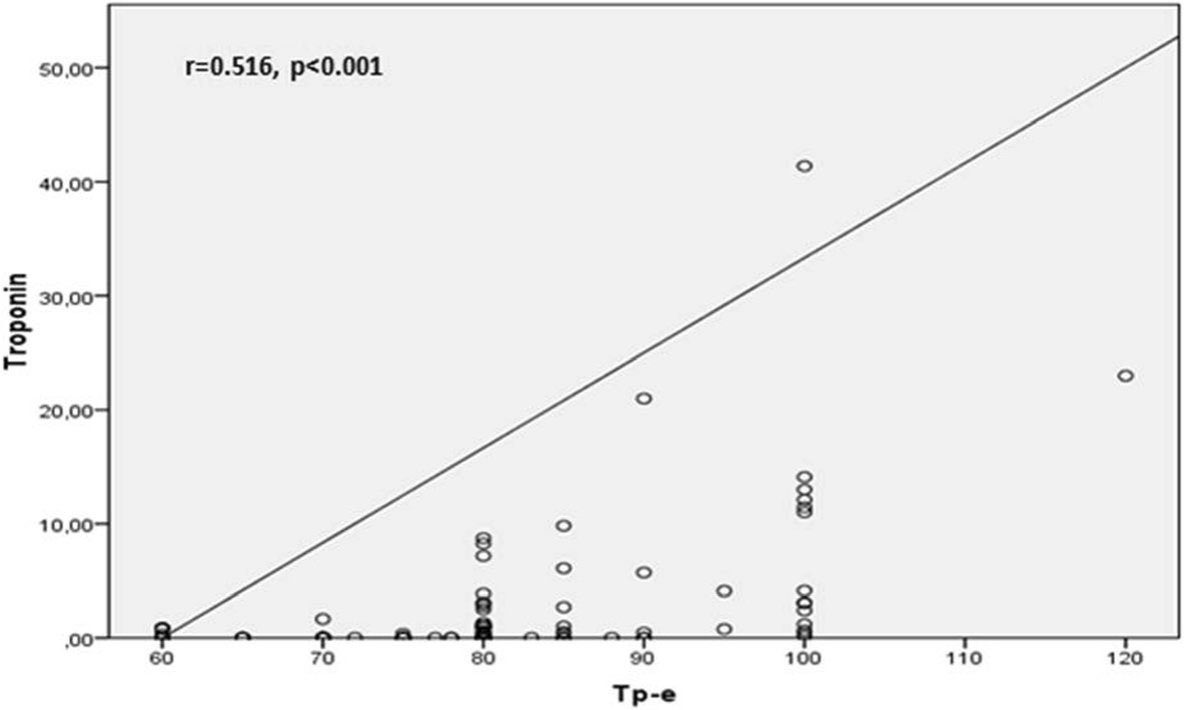
Correlation between troponin and peak and the end of the T wave (Tp-e) interval

**Fig. 2:**
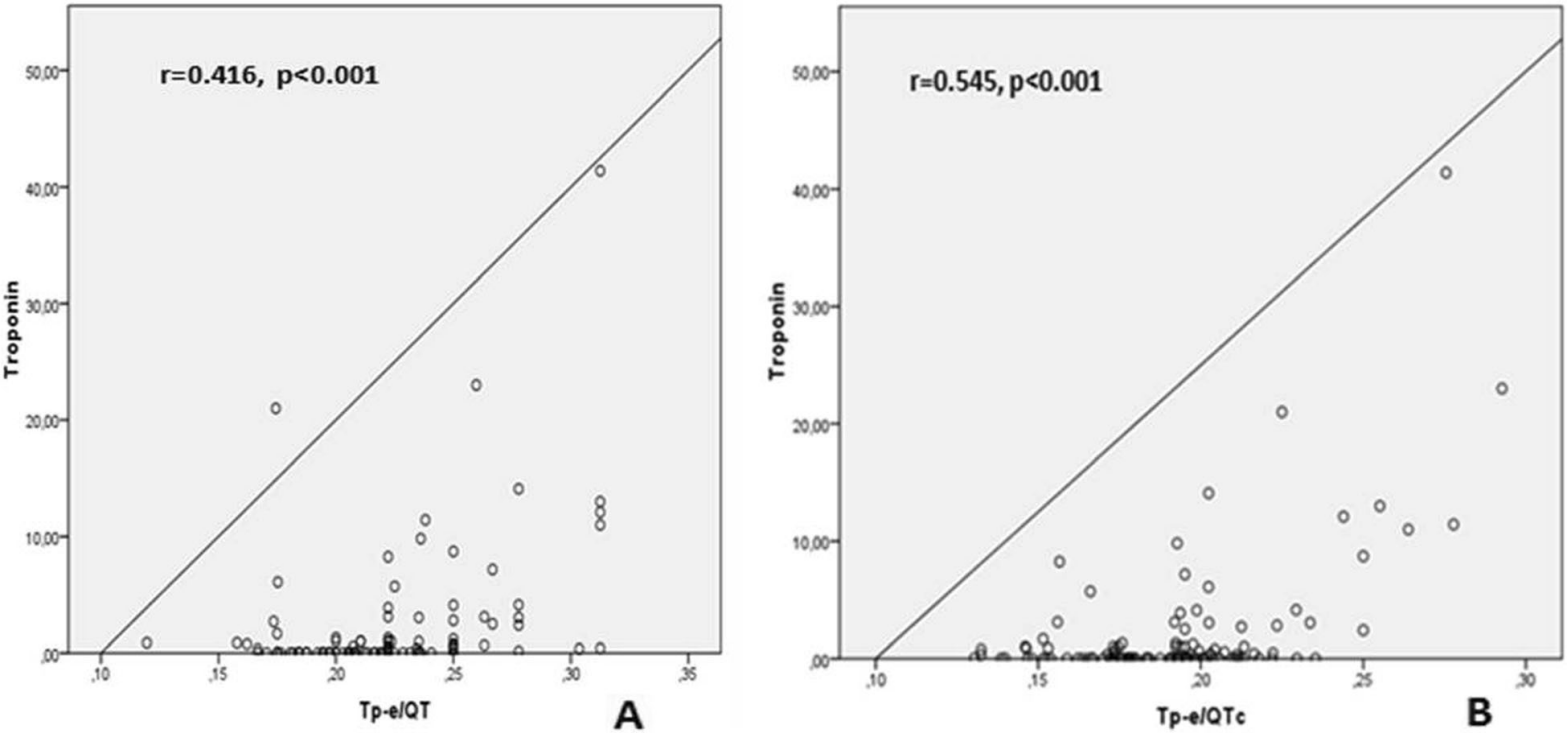
Correlation between troponin and Tp-e/QT ratio, and Tp-e/corrected QT interval (QTc) ratio. **a**:Correlation between troponin values and Tp-e/QT ratio, **b**:Correlation between troponin values and Tp-e/corrected QT interval (QTc) ratio

**Fig. 3:**
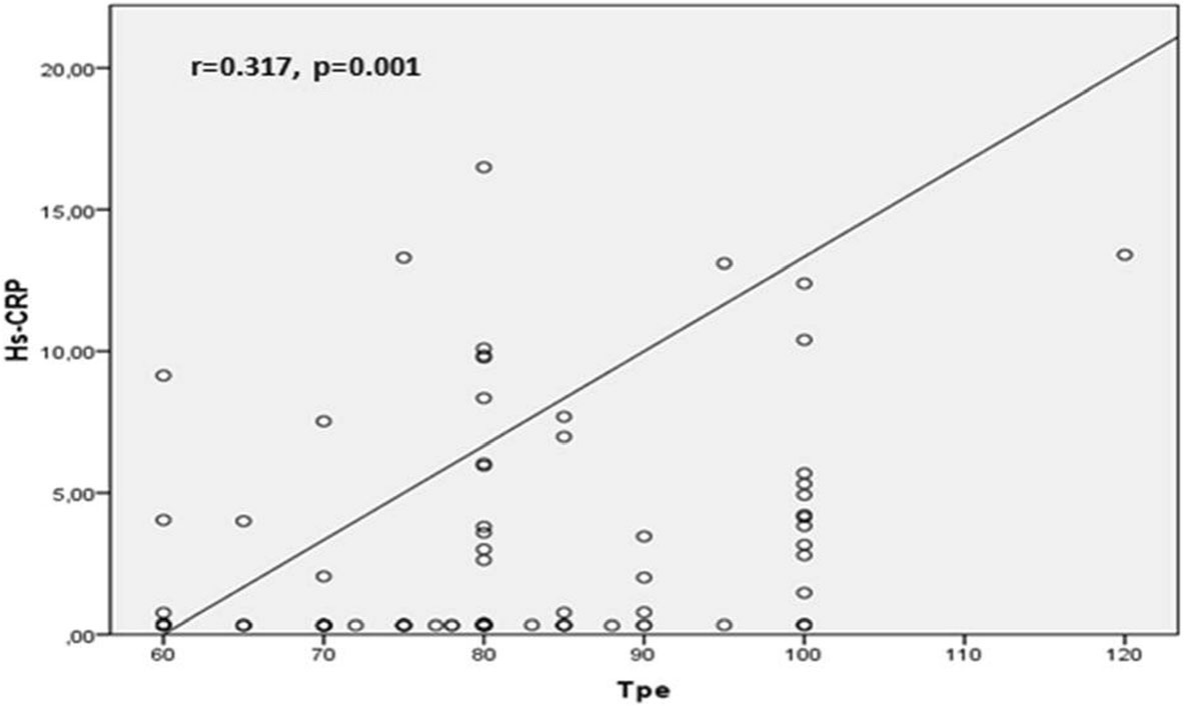
Correlation between high-sensitivity CRP (hs-CRP) and peak and the end of the T wave (Tp-e) interval

**Fig. 4:**
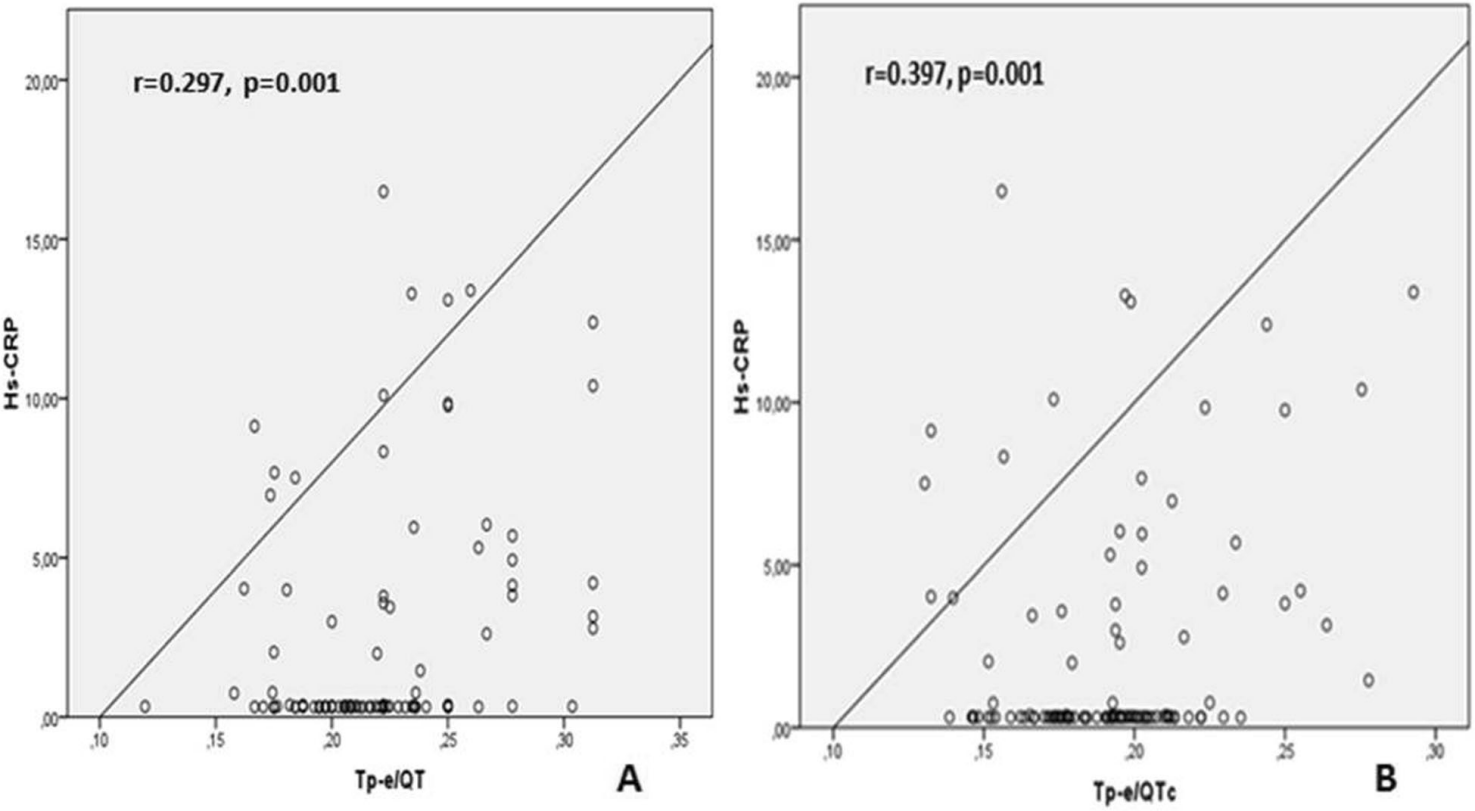
Correlation between high-sensitivity CRP (hs-CRP) and Tp-e/QT ratio, and Tp-e/corrected QT interval (QTc) ratio. **a**:Correlation between high-sensitivity CRP (hs-CRP) and Tp-e/QT ratio, **b**:Correlation between high-sensitivity CRP (hs-CRP) and Tp-e/corrected QT interval (QTc) ratio

Data are represented as mean values ±SD. LVEDD: *bpm* beats per minute, *Tp-e interval* T-peak to T-end interval; c = rate corrected value; B = corrected with Bazett’s formula;

Correlation analysis demonstrated a positive relation between Troponin-I and and Tp-e, Tp-e/QT and Tp-e/QTc ratios [(*r* = 0.516, *P* < 0.001), (*r* = 0.416, *P* < 0.001), (*r* = 0.545, *P* < 0.001) respectively] (Fig. 1, Fig. 2). Likewise, a positive correlation was also found between the HsCRP levels and Tp-e, Tp-e/QT and Tp-e/QTc ratios [(*r* = 0.317, *P* = 0.001), (*r* = 0.297, *P* = 0.001), (*r* = 0.397, *P* = 0.001) respectively] (Fig. 3, Fig. 4).

The mean duration of in hospital stay was 6 ± 2 days. Only 1 patient died because of cardiac arrest. We had 24 h and 48 h holter recordings of AM patients during hospitalisation and 4 of 56 AM patients had non sustained ventricular tachyarrhythmias. When we compared myocarditis patients with or without arrhytmia during hospitalisation, Tpe value,Tpe/QT and Tpe/QTc ratio values were all higher but there were not statistically significant.

## Discussion

The present study demonstrated that, in AM patients Tp-e interval was significantly prolonged, additionally Tp-e/QT and Tp-e/QTc ratios were also significantly increased in AM group. Furthermore, we demonstrated a positive correlation between HsCRP and CTn-I levels with these ECG indices.

A wide range of ventricular arrhythmias can occur in AM, including premature ventricular contractions and malignant VT. There are several hypothesis to obviate the underlying mechanism of VA’s in AM, but its exact mechanism has not been fully understood yet. Myocardial ion channel dysfunction and development of inflammation, particularly in electrically sensitive regions of myocardium, have been claimed as possible reasons of VA in AM [13]. Additionally, Pieroni et al. [13] implied regional slowing of action potential occurring due to fibrosis and secondary hypertrophy, as a possible cause of reentry circuits formation.

Clinical and instrumental characteristics of the patients were assessed for the arrhythmic risk prediction in AM [1]. Prolonged QT duration were observed in patients with fulminant myocarditis [14]. However, non-invasive and simple measurable ECG parameters,such as Tp-e interval, Tpe/QT and Tpe/QTc ratios have not been evaluated in patients with myocarditis before.

Recent studies have suggested that QTc and QT interval dispersion (QTd) are related with malignant VA’s [15, 16]. Furthermore Tp-e interval prolongation has also been associated with VA’s [17]. When compared to other myocardial cells, the duration of action potential lasts longer in the mid myocardial M cells, whereas in epicardial cells repolarization ends earlier. The peak of the T wave indicates the end of action potential in the epicaridium and the end of T wave symbolize the end of the action potential in the mid-myocardial region. Thus, Tp-e interval is an index of transmural DoR [18].

There are some factors such as body weight and heart rate that can also affect Tp-e interval. Tp-e/QT ratio, which is not affected by the variations in heart rate, was proposed as a more precise index than QT and QTc dispersion or TP-e interval, for demonstrating the DoR [8, 19]. Gupta et al. [9] claimed that Tp-e/QT ratio can be used as as an index of arrhytmogenesis even in the presence of short, normal, or long QT intervals, and additionally they indicated Tp-e/QT ratio, as a better marker of ventricular repolarization.

In the present study, we observed a prolongation in the Tp-e interval, and an increase in the.

Tp-e/QT and Tp-e/QTc ratios in patients with AM, when compared to healthy subjects. A significant positive correlation was found between the cTn-I levels and these ECG indices indicating that patients with an increase in these ECG markers may have higher risk of arrhythmias. Besides, patients with higher troponin levels may be followed for a longer time for arrhythmia monitorization.

## Study limitations

Our study has some limitations. Firstly, this is a resrospective study with a limited number of patients. Unfortunately *magnetic resonance imaging could not be used for the diagnosis of AM.* In addition, due to the diurnal variations in ECG parameters, usage of 24-h Holter ECG recordings would give more accurate data for assesing DoR. Although we used magnifying glass to measure ECG findings manually,; this type of measurement is less accurate than the automatic measurement. Therefore, a more comprehensive study is warranted to corroborate the predictive value of these ECG markers in patients with myocarditis.

## Conclusion

In conclusion, Tpe, Tpe/QT and Tpe/QTc ratios were all higher in acute myocarditis patients, which may indicate higher risk for arrhythmias, however further and larger-scale prospective studies are necessary to put-forth the prognostic importance of these repolarization dispersion indices in patients with myocarditis, for predicting arrhythmias.

## Data Availability

The datasets used and analysed during the current study are available from the corresponding author on reasonable request.

## Abbreviations

ALT: *Alanine transaminase*
AM: Acute myocarditis
AST: Aspartate aminotransferase
ECG: Electrocardiogram
hsCRP: *High-sensitivity C-reactive protein*
QTc: Corrected QT
Tpe: T peak-to-end
VF: Ventricular fibrillation
VT: Ventricular tachycardia
WBC: White blood cell
